# Perception of educational environment among undergraduate students of health disciplines in an Iranian university

**DOI:** 10.5116/ijme.5977.7129

**Published:** 2017-08-18

**Authors:** Arezou Farajpour, S. Mohammad Ali Raisolsadat, Samaneh S. Moghadam, Zahra Mostafavian

**Affiliations:** 1 School of Medical Education, Shahid Beheshti University of Medical Sciences, Iran; 2 Department of Surgery, Mashhad Branch, Islamic Azad University, Iran; 3 School of Medicine, Mashhad Branch, Islamic Azad University, Iran; 4 Department of Community Medicine, Mashhad Branch, Islamic Azad University, Iran

**Keywords:** DREEM, educational environment, evaluation, student perception, Islamic Azad University-Mashhad branch

## Abstract

**Objectives:**

This paper seeks to determine the perception of Medical, Nursing and Midwifery students about their educational environment and compare their perceptions in terms of disciplines, demographic attributes and academic level.

**Methods:**

In this cross-sectional study, Medical, Nursing and Midwifery students in Islamic Azad University, Mashhad, Iran, were selected using stratified random sampling method (N=378). They completed the standard Persian version of Dundee Ready Education Environment Measure (DREEM) questionnaire. Descriptive statistics, t-test and analysis of variance (ANOVA) were used to analyze data.

**Results:**

The mean score of DREEM was 106 ± 24.6. The mean scores in five domains of DREEM questionnaire including students’ perception of learning, perception of teachers, scientific abilities, students’ perception of educational environment and students’ perception of social conditions were 23±8, 23.4±6, 18±5.5, 25.5±7.7 and 15.8±4, respectively. In the first four domains (p=0.000, F=27.35), (p=0.000, F=9.9), (p=0.000, F=18.5), (p=0.000, t=18.7) and for total scores (p=0.000, F=22.77), the three disciplines were significantly different. Also, there was a significant difference between mean total score (p=0.021, t=2.3) and scores of students’ perception of learning (p=0.008, t=2.65) and social conditions (p=0.022, t=2.3) with respect to gender.

**Conclusions:**

According to these results, students tend to have a positive attitude towards their educational environment. The findings of this study are useful to identify areas in need of improvement by employing more specialized tools and planning for improvement.

## Introduction

An ideal and positive educational environment prepares students for professional life in the future. The educational environment should promote physical and mental status of students’ intellect.^1^^,^^2^ It encompasses all physical, mental and emotional conditions as well as socio-cultural factors that affect the development of learners in training institute.^3^ In 1988, the World Federation of Medical Education (WFME) considered the educational environment as an assessment area in medical training programs.^4^

The most important dimensions of the academic settings are teaching activities and teacher-student interactions in daily routines. Learning environment significantly affects the learning and behaviors of students.^5^^-^^7^ There is a strong relationship between learning environment and valuable components such as students’ satisfaction and success. The strengths and weaknesses of the learning environment should be identified to help change, adjust, and manage training programs with the aim of improving learning quality.^8^ The educational environment could be promoted and changed using appropriate evaluative methods.^9^^-^^11^ The Dundee Ready Education Environment Measure (DREEM) is an evaluative tool frequently used for the assessment of educational environments at Universities of Medical Sciences in various cultural settings, as shown by former studies.^4^^-^^11^ This questionnaire, designed by S. Roff and colleagues (1997) in the Delphi international panel at the University of Dundee, Scotland^12^^-^^14^, is used to diagnose curriculum problems, introduce efficient changes and compare real environment with a desired environment, which can provide valuable information for educational managers.^15^^,^^16^ The above questionnaire has been widely used by different institutes for various purposes such as inter-faculty and inter-student comparison,^18^ assessment of students’ perception of an ideal educational environment,^19^ comparison of ideal educational environment with existing environments and finally improvement of the educational environment.^17^ A growing number of studies have explored this subject around the world.^4^^-^^11^ Some Iranian studies have investigated educational environment in state-run universities.^21^^-^^28^ However, little attention has been paid to the educational environment in Islamic Azad University (IAU) as the main private organization offering medical training in Iran. Thus, this study intends to explore the educational environment at IAU of Mashhad, as one of the largest branches of IAU. This university offers three majors of Medicine, Nursing and Midwifery. This study, based on DREEM, attempts to determine and compare the perception of Medical, Nursing and Midwifery students about their educational environment. Further, students are compared in terms of demographic attributes and academic level.

## Methods

### Study design

This is a descriptive cross-sectional study conducted at Islamic Azad University of Medical Sciences, Mashhad, in 2015.

### Participants

The study population consisted of Medical (n=104), Nursing (n=138) and Midwifery (n=136) students who were selected using stratified random sampling. From the 400 questionnaires distributed, 378 were collected, with a response rate of 94.5%. The study was approved by Ethics Committee of Islamic Azad University of Medical Sciences, Mashhad branch. Prior to the study, researchers explained the objectives to participants, ensuring them about the anonymity, and voluntary nature of completing questionnaires.

### Data collection

The main data collection instrument was the DREEM questionnaire.^17^ Fallah and colleagues have evaluated the Persian version of DREEM, in terms of Cronbach's alpha (0.933), Kaiser-Meyer-Olkin (0.910), and the Bartlett (60936.37), and a significance level of p=0.001 was considered.^22^ The questionnaire consisted of two parts. The first part was about demographical information and the second part included 50 questions, which were scored on a 5-point Likert scale ranging from zero to four (4: strongly agree, 3: agree, 2: have no idea, 1: disagree, 0: strongly disagree). The items measure the perception and expectation of students about the educational environment of their university. Items divide into five categories: 1) students’ perception of learning (12 items); 2) students’ perception of course instructors (11 items); 3) students’ perception of their scientific competence (8 items); 4) students’ perception of the educational environment (12 items); and 5) students’ perception of social conditions (7 items).

Among questions, nine (4, 8, 9, 17, 25, 35, 39, 48 and 50) are negative and thus must be coded reversely. The maximum score of the DREEM questionnaire is 200. Higher scores indicate more positive and convenient educational environment. To identify strengths and weaknesses of the educational environment under study, items with an average score of 3.5 and higher, items with an average score of 2 and lower, and items with an average score of 2-3 are considered as strengths, problematic areas, and areas in need of improvement, respectively.^17^

### Data analysis

After collecting questionnaires completed by students anonymously, data was entered in SPSS 20 for analysis. In addition to descriptive statistics, analytical statistical tests were carried out using independent samples t-tests and one-way analysis of variance (ANOVA) at a significance level of p<0.05.

## Results

All participants were in the age range of 18-40 years (mean=22.1±2.3). In terms of gender, 298 participants (80%) were female. The number of students in each major, their educational levels, gender and marital status are shown in Table 1. The mean score of DREEM was 106 ± 24.6. The mean score, total score and their implication for each subscale is listed in Table 2. The mean score of each subscale and their inter-major comparisons are shown in Table 3.

**Table 1:** Demographic of students

Students	N	Educational level		Marital status		Gender	
		pre-clinical	clinical	married	single	female	male
		n (%)		n (%)		n (%)	
Medicine	104	56 (54)	48 (46)	16 (15.4)	88 (84.6)	80 (77)	24 (23)
Midwifery	138	84 (61)	54 (39)	45 (32.6)	93 (67.4)	138 (100)	0 (0)
Nursing	136	75 (55)	61(45)	41 (30)	95 (70)	80 (58)	56 (42)
Total	378	215 (57)	163 (43)	102 (27)	276 (73)	298 (80)	80 (20)

The mean score of students’ perception of learning was 23±8 with Medical students having significantly more negative attitudes (p=0.000, F=27.35). The mean score of students’ perception of instructors was 23.4±6 with students of Midwifery having significantly more positive attitudes towards their instructors (p=0.000, F=9.9). The mean score of students' perception of their scientific competence was 18±5.5, with Medical students having significantly negative attitudes about their academic abilities (p=0.000, F=18.5). The mean score of students’ perception of the educational environment was 25.5±7.7 with Medical students (average score = 22±7) holding significantly low view of educational environment (p=0.000, t=18.7). The mean score of students’ perception of social conditions was 15.8±4 and three majors of Medicine, Nursing, and Midwifery were significantly different in this regard (p=0.000, F=22.77).

**Table 2:** Scores of each subscale based on score analysis guide

Subscales	Score analysis guide	Average score
Domain 1. student’s perception of learning Max score:48	0-12: very weak 13-24: negative attitude towards education 25-36: positive perception 37-48: effective education	23±8
Domain 2. student’s perception of the course instructors Max score:44	0-11: very weak 12-22: needs relearning 23-33: moving in the right direction 34-44: distinguished professors	23.4±6
Domain 3. student’s perception of their scientific competence Max score:32	0-8: general feeling of inadequacy 9-16: high negative dimensions 17-24: positive feeling 25-32: confidence	18±5.5
Domain 4.student’s perception of the educational environment Max score:48	0-12: terrible environment 13-24: huge modifications are required 25-36: more positive attitude 37-48: general positive feeling	25.5±7.7
Domain 5. student’s perception of social circumstances Max score:28	0-7: highly undesirable 8-14: not a good place 15-21: not so bad 22-28: desirable social conditions	15.8±4
Total: 200	0-50 completely unsatisfactory 51-100 denotes an environment with many problems 101-150 generally conveys a positive attitude 151-200 a perfect environment	106 ± 24.6

According to the results, the mean score of DREEM showed a significant difference between male and female students in all majors (p=0.02, t=2.3). Further, the mean total score and the mean score of students’ perception of learning and social context were higher in female students, though no significant difference was observed in students’ perception of their scientific competence (Table 4).

Table 5 shows data derived from the clinical and pre-clinical students. A comparison of the mean score of DREEM and the mean score of areas under study in both clinical and pre-clinical environments did not show any significant difference. However, the mean total score of Nursing students was significantly different (p=0.042, t=2.05) from students of Medicine and Midwifery. In other words, the mean DREEM score of nursing students in clinical environments was significantly higher than students in pre-clinical setting.

## Discussion

The issue of quality improvement and measures aimed at optimization of medical training is of paramount importance in the Faculties of Medicine. This study intended to identify areas of weaknesses in need of revision, change, and reformation using the DREEM tool so that all students were able to access to desirable and convenient educational environment without any waste of time, energy and capital.

In this study, the mean score of DREEM was 106±24.6, which reflected a relatively positive attitude. In some studies conducted in different state-run Iranian Medical Universities, the reported score was as follows: Zanjan University of Medical Sciences^26^:100.26, Hormozgan University of Medical Sciences^21^:99.6, Isfahan School of Dentistry^25^: 99.75, Yazd University of Medical Sciences^27^: 110 and Gilan University of Medical Sciences^28^:98. Public universities in Iran are funded by the state and their students do not pay a tuition fee. Given the similarity of the scores from this study with public universities above, it seems that students at this private institution enjoy the same level of satisfaction as the students attending state-run universities. This is in contrast to the findings of Dashputra and colleagues which revealed that students of private universities scored significantly higher in comparison to state-run public universities, suggesting that their private institutions were more student-centered, intimate, and convenient.^29^

The DREEM score in the present study was consistent with those reported in other countries such as state universities of Pakistan (105),^30^^,^^31^ Ankara University (117.6),^5^ King Saud University (89.9),^32^ Trinidad (109.9),^33^ Sri Lanka (108),^34^ India(107.44),^29^ Umm Al-Qura University (100),^11^ and Canadian Memorial Chiropractic College (97).^35^ On the other hand, the mean total score was 126.3 for newly-established medical school of India,^29^ 137.3 for Monash University of Australia,^9^ 135.37 for Victoria University of Melbourne,^16^ 139 for Dundee Faculty of Medicine,^32^ 130 for Nepalese Faculty of Medicine^17^ and 135.4 for Arab Golf University in Bahrain.^36^ The disparity of scores reported in different universities suggests that the educational environment is largely influenced by the curriculum adopted in that university. As such, higher DREEM scores indicate that curriculum development was based on modern medical education principles and training whereas scores smaller than 120 depict a traditional education system as mentioned in other studies.^23^^,^^30^

This disparity of DREEM scores in various universities around the world suggests that all educational environments have certain strengths and weaknesses. Considering that the mean total score is never beyond 143, it seems that students often have requests and expectations in all majors and educational environments. The results of this study revealed relatively positive attitudes in all areas under study except for the first subscale (learning subscale: 23±8). Given the variety of curriculums and contexts investigated in different studies, it is not possible to find a definite pattern because the lowest global score reported in studies related to students’ perception was inconsistent.^30^^,^^37^ Imanipour and colleagues maintain that the main reason for achieving low score in learning areas is the disregard of lifelong learning strategy in the traditional education, which could be achieved by developing metacognition capabilities in educational trends such as critical thinking and problem solving.^24^

**Table 3:** Comparison of average score for each major, mean total score of different majors and their level of significance

Domains	Medicine	Midwifery	Nursing	Total students	F	p-value	Tukey ≤ 0.05
Domain 1: student’s perception of learning Max score:48	18.5±7	25.5±6.9	24±7.8	23±8	27.35	0.0001	medicine–midwifery medicine –nursing
Domain 2: Student’s perception of the course instructors Max score:44	22±5.8	25±6	22.6±6.4	23.4±6	9.9	0.0001	medicine–midwifery medicine –nursing
Domain 3: Student’s perception of his/her scientific competence Max score:32	15.4±5.4	18.9 ±5	19.35±5.4	18±5.5	18.5	0.0001	medicine–midwifery medicine–nursing
Domain 4: Student’s perception of the educational environment Max score:48	22±7	27.9±7	25.6±7.5	25.5±7.7	18.7	0.0001	medicine–midwifery medicine–nursing nursing-midwifery
Domain 5: Student’s perception of social conditions Max score:28	15.5±4	16.4±4.3	15.6 ±4.45	15.8±4	1.69	0.186	-
Total	93.7±23.4	114±23	107±23	106±24.6	22.77	0.0001	medicine–midwifery medicine –nursing nursing-midwifery

**Table 4:** Gender-based comparison of the average score of subscales

Domains	Female	Male	*t*	p-value
Domain 1: Student’s learning perception Max score:48	23.55±7.8	20.9±8	2.65	0.008
Domain 2: Student’s perception of the course instructors Max score:44	23.75±6	22.17±7	1.77	0.079
Domain 3: Student’s perception of his/her scientific competence Max score:32	18±5	18±6	-0.129	0.89
Domain 4: student’s perception of the environment Max score:48	25.8±7.6	24±8	1.827	0.068
Domain 5: student’s perception of social circumstances Max score:28	16±4	15±4	2.3	0.022
Total	107.4±24.5	100.3±24.7	2.3	0.021

A comparison of the scores of Medicine, Nursing, and Midwifery students showed that Medical students scored lowest on mean total score of DREEM (93.7±23.4), followed by students of Nursing (107±23) and Midwifery (114±23), and there was a significant difference among these majors. Likewise, Brown and colleagues found that Australian DREEM scores were significantly different in Medicine, Midwifery, Physiotherapy, Nutrition, Emergency, Radiology, and Pharmacy students.^9^ They found the lowest score belonged to Pharmacy (133) and the highest average scores were observed in Nutrition and Diet Therapy (145.5) and Emergency (143.4), respectively. In contrast, Montazeri and colleagues found no differences between the mean total scores in different majors of Nursing, Midwifery, Anesthesia, Operating Room, Radiology and Laboratory Medicine in Yazd University of Medical Sciences, Iran.^27^ These findings may suggest that these differences indicate the extent of curriculum success among different departments.

This study demonstrated a significant difference between the average scores of male and female students in different subscales with the latter scoring higher in subscales of learning and social conditions. Studies in Saudi Arabia^37^ and Pakistan^30^ have reported a positive and significant relationship between DREEM score and students’ gender, finding that female students score higher in mean total and subscales. In Australia, the DREEM score of females was 138.8, which was significantly greater than that of males (132.3).^9^ This was consistent with the results of a study in UK which used the same curriculum. Strong evidences regarding the different learning styles of males and females may justify the above differences. Some experts argue that it is stronger interpersonal communicative skills of women which makes them more appreciative of the positive perception of educational environments.^30^ On the other hand, the greater number of female students in medical sciences and medical professions in recent decades may have directed educational plans and materials towards the learning requirements of females. Other effective factors may be social conditions and job opportunities in Iran. We could also speculate that since the main concern of men could be to earn income from other resources, they pay less attention to their academic major compared to women. Studies in the Middle East, India, and Sri Lanka have reported different results, which is often a variable of socio-cultural elements of communities under study.^29^^,^^30^^,^^34^^,^^42^^-^^44^

**Table 5:** Comparison of DREEM score in clinical and pre-clinical students

Disciplines	Domains	Pre –clinical	Clinical	*t*	Sig
Medicine	Domain 1:Student’s perception of learning Max score:48	17.9±6.5	19±8	0.97	0.331
	Domain 2:Student’s perception of the course instructors Max score:44	21.9±5.5	22±6	0.19	0.848
	Domain 3:Student’s perception of his/her scientific competence Max score:32	15.4±4.5	15.4±6.2	0.06	0.95
	Domain 4: Dtudent’s perception of the educational environment Max score:48	21.8±7.4	22.4±7	0.49	0.62
	Domain 5:student’s perception of social conditions Max score:28	15.7±4	15.3±4	-0.433	0.66
	Total	92.7±21.3	94.8±25.8	0.45	0.651
Midwifery	Domain 1:student’s perception of learning Max score:48	25.5±6.6	25.5±7.3	-0.025	0.98
	Domain 2:student’s perception of the course instructors Max score:44	25.3±6	25±6.4	-0.153	0.87
	Domain 3:student’s perception of his/her scientific competence Max score:32	19±5.5	18.6±4	0.555	0.58
	Domain 4: student’s perception of the educational environment Max score:48	27.7±7.7	28.3±6.8	0.467	0.64
	Domain 5:student’s perception of social conditions Max score:28	15.9±4.7	17±3.7	1.53	0.12
	Total	113.6±24.4	114.7±21.5	0.263	0.79
Nursing	Domain 1:student’s learning perception Max score:48	22.4±8	25.6±7	2.42	0.017
	Domain 2:student’s perception of the course instructors Max score:44	21.9±6	23.4±6.6	1.43	0.15
	Domain 3:Student’s perception of his/her scientific competence Max score:32	18.7±5.3	20±5.4	1.48	0.14
	Domain 4: Student’s perception of the educational climate Max score:48	24.6±7.5	27±7	1.78	0.076
	Domain 5:Student’s perception of social conditions Max score:28	15.7±4.4	15.4±4.5	-0.416	0.67
	Total	103±24.5	111.5±21	2.05	0.042
Total	Domain 1:Student’s perception of learning Max score:48	22.4±7.7	23.7±8	1.56	0.118
	Domain 2:Student’s perception of the course instructors Max score:44	23±6	23.6±6.5	0.6	0.545
	Domain 3:Student’s perception of his/her scientific ability Max score:32	18±5.4	18±5.6	0.4	0.68
	Domain 4: Student’s perception of the educational environment Max score:48	25±8	26±7	1.2	0.22
	Domain 5:Student’s perception of social conditions Max score:28	15.8±4	16±4	0.33	0.73
	Total	104.6±25	107.6±24	1.2	0.233

The results of the present study showed that with the exception of nursing students, the hike in DREEM scores in both pre-clinical and clinical education was not significant. In the study of Menaka in Sri Lanka,^34^ no significant difference was observed in the positive perception of freshmen. The results of some studies undertaken by Imran (2015)30 in Pakistan, Al-Ayed (2008)^38^ in King Saud University and Palés and colleagues (2015)^39^ in five Spanish medical schools demonstrated a significantly higher mean total score in pre-clinical students compared to clinical students. It is expected that a professional educational program is able to motivate creativity and self-efficacy in learners so that they can continue learning during their academic education. As a result of engagement in actual professional environments and application of theoretical educations, students in this research had more positive perception of their educational environment. Shankar and colleagues explored students’ perception of the educational environment in Xavier University of Netherlands using DREEM, finding that the mean score of students’ perception was 131.79.^40^ Researchers found that they needed to receive ongoing feedbacks from students to optimize the curriculum and implement student-centered and merging strategies. Accordingly, after making some changes in teaching and learning dimensions, this study was repeated in 2014 and a DREEM mean total score of 151.32 was achieved. The results showed improvement in all areas caused by to the adoption of an integrated curriculum.^41^ Studies in UAE,^42^ Pakistan^43^^,^^44^ and Chile^4^ have achieved similar results using modern and innovative curriculums. Still, more analytical studies are required to clarify the origins and causes of these differences and experimental studies to provide practical solutions to promote more positive perception in students.

## Conclusions

The results of this study reveal the overall positive attitude of students, implying that the positive dimensions of the educational environment outweigh its negative aspects. This study shows that IAU University, similar to other universities in the region, may utilize a traditional education method, which needs to be boosted using modern educational approaches. However, the findings help us to identify areas in need of improvement and revision, which could be investigated using more specialized instruments to make necessary planning for improvement.

### Conflict of Interest

The authors declare that they have no conflict of interest.
